# Medication related problems among ambulatory patients with chronic kidney disease at St. Paul’s Hospital Millennium Medical College, Addis Ababa, Ethiopia

**DOI:** 10.1371/journal.pone.0278563

**Published:** 2022-12-01

**Authors:** Eshetu Shiferaw Legesse, Oumer Sada Muhammed, Leja Hamza, Beshir Bedru Nasir, Teshome Nedi

**Affiliations:** 1 Yekatit 12 Hospital Medical College, Addis Ababa, Ethiopia; 2 Department of Pharmacology and Clinical Pharmacy, School of Pharmacy, College of Health Sciences, Addis Ababa University, Addis Ababa, Ethiopia; 3 St. Paul’s Hospital Millennium Medical College, Addis Ababa, Ethiopia; Al-Jouf University College of Pharmacy, SAUDI ARABIA

## Abstract

**Background:**

Medication related problem (MRP) is an event occurring, as a result, the medication therapy that actually or potentially interferes with desired health outcomes. Evidences reported that the prevalence of MRPs may result in a high burden of morbidity and decrease patients’ quality of life. The problem is more significant among patients with chronic kidney disease (CKD) as a decline in kidney function and increase number of medications required to treat kidney disease and its complications. Thus, this study aimed to assess MRPs and its associated factors among patients with chronic kidney disease.

**Method:**

Hospital-based cross-sectional study was conducted among 248 adult ambulatory patients with CKD (stage 1–4) at St. Paul’s Hospital Millennium Medical College. Data were collected through patient interview and medical chart review from 1^st^ of June to 30^th^ of August 2019. MRPs were identified based on the standard treatment guidelines. Cipolle MRPs classification was used to classify the MRPs and Micromedex^®^ was used as drug interaction checker. Binary logistic regression was utilized to identify the associated factors and p value <0.05 was considered as statistically significant.

**Result:**

A total of 325 MRPs were identified from 204 (82.3%) study participants giving 1.6 MRPs per participant. One MRP was identified among 114 (55.9%) patients while two MRPs were identified among 64 (31.4%). The most common class of MRPs were need additional drugs 114 (35.1%) followed by non-compliance 54 (16.6%), unnecessary drug therapy 46 (14.2%) and dose too low 46 (14.2%). The two most common reasons for non-compliance were unaffordability of drugs 26(48.1%) and the lack of patient understanding about drug taking instruction 10 (18.5%). The study showed that only occupation (AOR = 5.2, 95% CI: 1.292–21.288, P = 0.020) and angiotensin converting enzyme inhibitor use (AOR = 6.6, 95% CI: 2.202–19.634, P = 0.001) had an association with the occurrence of MRPs.

**Conclusion:**

The prevalence of MRPs among ambulatory patients with CKD was high and need of additional drug therapy was the commonest MRPs.

## Introduction

Non-communicable diseases are the major concerns in the 21^st^ century world-wide [1]. Chronic kidney disease (CKD) is among the leading non-communicable diseases of public health concern [2]. It is defined as a reduction in the Glomerular Filtration Rate (GFR) and urinary abnormalities or a structural abnormality of renal tract [3]. It is also explained by any one of the following findings: pathologic kidney abnormalities, persistent proteinuria, other urine abnormalities, *e*.*g*., renal hematuria, imaging abnormalities and *eGFR*<60 mL/min/1.73 m^2^ on two occasions separated by ≥90 days and that is not associated with a transient, reversible condition such as volume depletion [4]. The disease commonly affects the proximal tubules and the interstitium in turn giving rise to characteristic, recognizable histopathological and clinical features. In clinical evidences the disease is manifested by tubular proteinuria, usually ß2-microglobulinuria, and the absence of hypertension and edema [5]. Due to this effect, the incidence and prevalence of CKD in both developed and developing countries have been considered as public health problems [6].

Furthermore, CKD was associated with alteration in the pharmacokinetic properties of a range of drugs especially renal-excreted drugs. This alteration may include changes in drug bioavailability, protein binding level, drug distribution and elimination. Unfortunately, this pharmacokinetics change will make patients vulnerable to medication related problems (MRPs) and other co-morbidities [7].

As kidney functions begins to decline, additional medications to prevent and manage CKD complications are unavoidable, including treatment for mineral and bone disorders, anemia, hyperlipidemia and cardiovascular complications. So it is not amazing that by the time patients reach stage 5 CKD on dialysis that need an average of 10–12 medications. The large number of medications and the presence of renally altered drug disposition put such a patient population at high risk for having MRPs [8]. In general, the above factors increase the patients’ risk of having MRPs [9].

MRP is defined as “an event occurring, as a result, the medication therapy that actually or potentially interferes with desired health outcomes” [10]. It has been evident that the prevalence of MRPs may result in high burden morbidity and decrease patients’ quality of life in different clinical settings [11].

Since the occurrence of MRPs increase in CKD patients as their clinical condition become worsen, with more associated cardiovascular risk factors and more treatments to stabilize their clinical condition, health professionals should be aware regarding such problems and ensure the adoption and implementation of a preventive strategy [12].

Currently, MRPs are of major concern in the health care delivery system because of increased burden in cost, morbidity and mortality. It was also known to be associated with a lower quality of life [13]. A study conducted in India indicates that the cost of drug-related morbidity and mortality exceeded $177.4 billion in 2000 of total costs, followed by long-term-care admissions, which accounted for 18% ($32.8 billion) [14]. A study in Singapore showed that 71.9% of MRPs resulted in admissions [15], while from a prospective multicenter study among 13,000 unplanned admissions, 714 (5.6%) were medication related [16]. A study in New York also showed that MRPs were associated with mortality, morbidity, and lower quality of life [13].

Little is known about the prevalence and specific predictors of MRPs in patients with CKD residing in Ethiopia. Therefore, this study aimed to assess the extent of MRPs and the associated factors among patients with CKD in St. Paul’s Hospital Millennium Medical College, Ethiopia.

## Materials and methods

### Study setting

The study was conducted at St. Paul’s Hospital Millennium Medical College, which is located in Addis Ababa, Ethiopia. The hospital was established in 1968, while the medical school was opened in 2007 [17]. Currently, it is one of the tertiary public hospitals in Ethiopia with more than 400 beds that provide services for different areas of ambulatory and admitted patients coming from different corners of the country. It has different clinics which provide outpatient services where patients with specific chronic diseases are referred for follow-up and routine health services for patients in the capital city and regional states of Ethiopia. The renal clinic is one of the specialty clinics that provides service to patients with CKD and hypertension at this hospital.

### Study design and period

A hospital-based prospective cross-sectional study was conducted among ambulatory patients with CKD who came to St. Paul’s Hospital Millennium Medical College, Addis Ababa, Ethiopia from the 1^st^ of June to 30^th^ of August 2019. The study population consisted of ambulatory adult patients diagnosed with CKD and on medication therapy and who had follow-up at St. Paul’s Hospital Millennium Medical College during the study period.

### Eligibility criteria

All ambulatory adult patients with CKD (stages 1–4), those who were on medication therapy at St. Paul’s Hospital Millennium Medical College during the study period and who were willing to participate in the study were included. The stage of kidney disease was categorized using eGFR, calculated based on the serum creatinine levels using the MDRD formula. Patients who were post-renal transplant, on hemodialysis and who had cognitive impairment were excluded.

### Sample size determination and sampling techniques

The number of patients to be involved in the study was determined by using the single population proportion formula:

$$n=\frac{Z^{2}{}_{a/2}P(1‐P)}{d^{2}}$$

Where

**n** = Minimal sample size required.

**P** = Estimated prevalence of MRPs in CKD.

**Z**^**2**^_**a/2**_ = Standard normal deviate at 95% confidence interval corresponding to 1.96

**d** = Absolute error between the estimated and true population prevalence of CKD of 5%.

By using P value of 50%, the calculated sample size was 384. The expected number of source populations in the study period (N), based on the average number of patients coming to the renal sites during the study period gave us 540.

Hence, the *corrected sample size* (***nN/n+N***) **was 225**. By adding 10% contingency, the final sample size used in this study was **248**.

A systematic random sampling technique was used to recruit samples for the study on each day of the data collection process. The actual sampling fraction (k^th^) was calculated by dividing the total number of source population attending the renal clinic during the study period (540) by the corrected sample size (248). The first patient was selected randomly, then every other patient was selected from the patient registration list and interviewed after the physician visit and respective medical record was reviewed on the same day after the interview.

### Data collection process

The data collection was conducted by three nurses, one clinical pharmacist and the principal investigator. One-day training was given to all data collectors before data collection on how to interview patients and use the abstraction format to gather information. Data collectors completed an interviewer-administered questionnaire which aimed at collecting socio-demographic details and medication history data from the patient. Then the investigator reviewed the patients’ medical records. The data collection was carried out in two steps. The first was a patient interview used to obtain socio-demographic details such as age, sex, marital status, level of education, occupation, level of income and patient status on cigarette smoking and alcohol intake. Past medical history, medication history and MRPs reported by the patient and other aspects of patient related risk factors associated with MRPs were collected. The second step was medication chart review which was used to collect medical history, physical examination notes, results of laboratory and diagnostic tests, working diagnosis and treatments. In addition, the presence of co-morbidities, number of co-morbidities, and presence of complications, stage of CKD and type as well as the number of drugs were reviewed in this section.

Identification and classification of MRPs using appropriate guidelines was assessed by 2 clinical pharmacists through review and analysis of all medication orders, administration sheets, laboratory and diagnostic test results and working diagnosis. The identification of MRPs and principles of medication use in CKD were based on evidence-based clinical guidelines and standards of practice like: KDIGO 2017; Clinical Practice guideline Update for the Diagnosis, Evaluation, Prevention, and Treatment of Chronic Kidney Disease, National Kidney Foundation [18], Guidelines on Clinical and Programmatic Management of Major Non-Communicable Diseases 2016 [19], and Ethiopian Standard Treatment Guideline for General Hospital 2014. For every participant, we defined a MRP as a single binary variable indicating the presence or absence of the identified MRP. Drug–drug interactions were checked using IBM Micromedex and Medscape online clinical information software.

#### Data quality assurance

The data abstraction format was assessed by an expert physicians in the field of nephrology for completeness and clarity. A pre-test was done among 13 patients before the actual data collection to check for the uniformity and understandability of the questionnaire. Regular supervision and follow up were conducted during data collection the principal investigator also checked for completeness and consistency of the collected data on daily basis.

### Data analysis

After the data was checked for completeness, it was entered into Epi Info 7 software version 7.1.4 and the analysis was conducted by SPSS v 25. Descriptive statistics were used to summarize frequencies and percent proportions. Multivariable binary logistic regression analysis was used to assess association of the independent variables with MRPs after univariable analysis (p<0.2) to control confounders. Then a result with a p value <0.05 was considered significant.

### Ethical consideration

Ethical clearance was obtained from the Ethical Review Committee of School of Pharmacy, Addis Ababa University (EBR/SOP/55/03/2019) and Ethical Review Board of St. Paul’s Hospital Millennium Medical College (PM.23344). An official letter was written by the Ethical Review Board of St. Paul’s Hospital Millennium Medical College, to the outpatient renal clinics. Written and verbal consent from the patients was obtained and confidentiality of the information was assured by using code numbers rather than personal identifiers.

## Results

### Socio-demographic characteristics of the study participants

In this study, a total of 248 patients were included. The majority 132(53.2%) of them were males. The mean age of the study participants was 53.01 ± 15.2 years. More than half (59.3%) of the patients were in the age group of 18–59. Among the patients, 37.1% has completed secondary education and 44.8% were employed. Moreover, 5.6% and 4.4% of the participants use alcohol and khat, respectively (Table 1).

**Table 1:** Socio-demographic characteristic of CKD at St. Paul’s Hospital Millennium Medical College, Addis Ababa, Ethiopia, June-August, 2019.

Variables	Category	Numbers	Percent (*%*)
Gender	Male	132	53.2
	Female	116	46.8
Age	18–59	147	59.3
	≥60	101	40.7
Marital status	Single	63	25.4
	Separated	29	11.7
	Married	135	54.4
	Divorced	10	4.0
	Widowed	11	4.4
Educational level	Cannot read and write	32	12.9
	Primary	80	32.3
	Secondary	92	37.1
	College and above	44	17.7
Occupation	Unemployed	100	40.3
	Employed	111	44.8
	Retired	37	14.9
Social drug use	Alcohol use	16	5.6
	Cigarette smoking	6	2.4
	Khat chewing	11	4.4

### Clinical characteristics of study participants

More than half (53.6%) of the study participants were in stage 4 and about 86.7% of the patients had at least one co-morbidity in which hypertension (37.5%) and diabetic mellitus (17.7%) were the commonest ones. Among the participants, 64.5% had one or more of which anemia (56.9%) and hyperphosphatemia (46.9%) were the most frequently reported complications. A total of 922 medications were used, with a mean of 3.72 ± 1.4 per patient (Table 2).

**Table 2:** Clinical characteristics of CKD at St. Paul’s Hospital Millennium Medical College, Addis Ababa, Ethiopia, June-August, 2019.

Variables	Category	Number	Frequency (%)
Stage of CKD	Stage 1	5	2.0
	Stage 2	19	7.7
	Stage 3	91	36.7
	Stage 4	133	53.6
Co morbidities	Present	215	86.7
	Absent	33	13.3
Specific co morbidities*	HTN	81	37.5
	DM	38	17.7
	CVD	14	6.5
	DM nephropathy	24	10.9
	HTN nephropathy	33	14.9
	Glomerulonephritis	4	1.6
	Hepatitis	13	6
	Uropathy	3	1.2
	HTN nephrosclerosis	12	5.6
	TB	3	1.2
	HIV	4	1.6
	Others**	7	3.2
Complication***	Present	160	64.5
	Absent	88	35.5
Specific complication	Anemia	91	56.9
	Osteodystrophy	28	17.5
	Hyperkalemia	3	1.9
	Hyperphosphatemia	75	46.9
	Neuropathy	21	13.1
No of medication per patients	≤3	86	42.2
	≥4	118	57.8

* 13 patients had two and 4 patients had 3 complications respectively

** Others: Community acquired pneumonia, urinary tract infection, gout, peptic ulcer disease, asthma, Parkinson

***One patient had 4 complications, 4 patients had 3 complications and 21 patients had 2 complications.

CVD: Cardiovascular diseases, DM: Diabetes mellitus, HTN: Hypertension, TB:Tuberclosis

**Fig 1:**
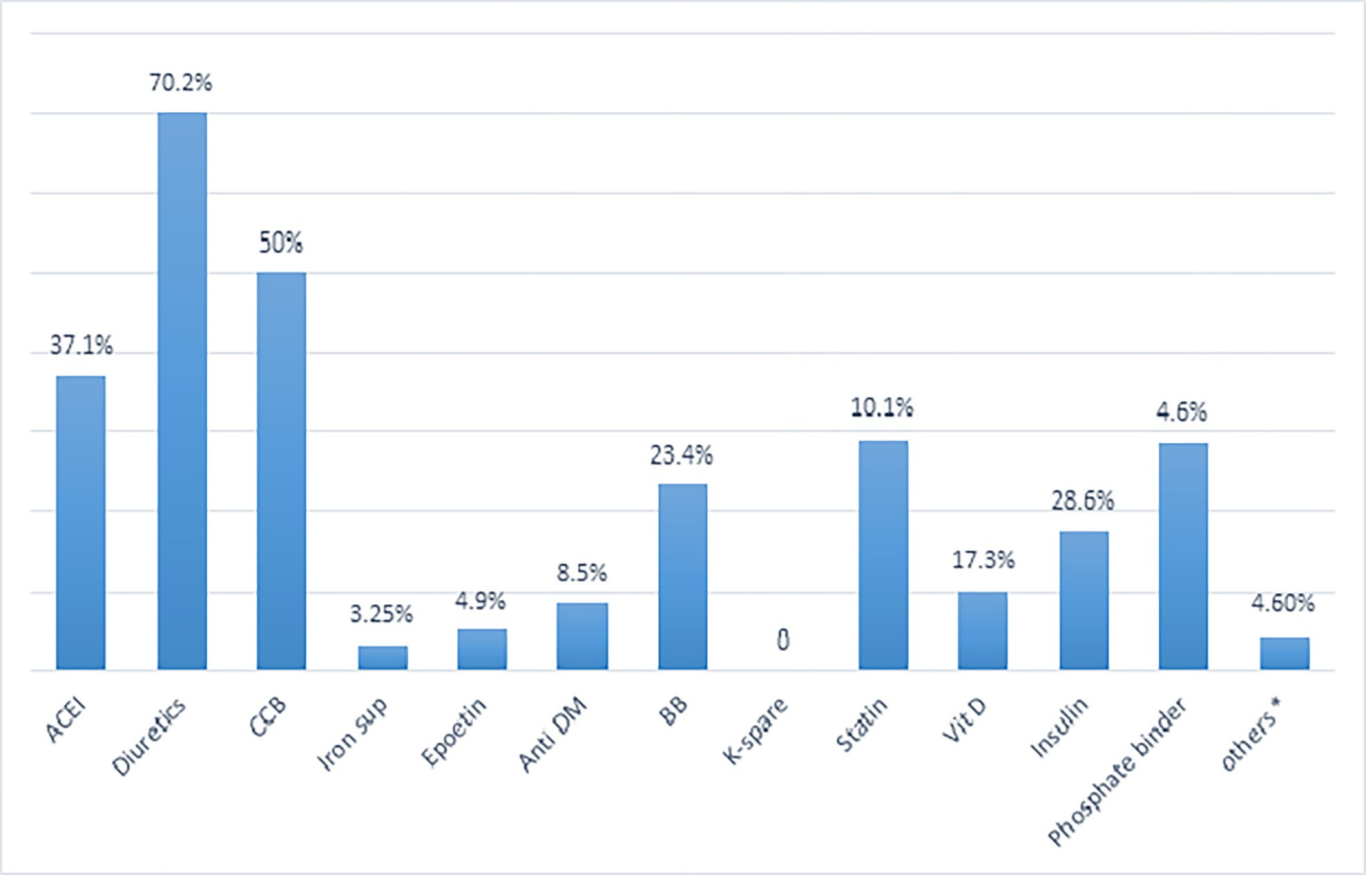
Frequently prescribed drug class of CKD at St. Paul’s Hospital Millennium Medical College, Addis Ababa, Ethiopia, June-August, 2019. * Others: aspirin, anticoagulant, antibiotics, antiparkinson, proton pump inhibitors, histamine 2 antagonists, short acting beta agonists, allopurinol, anti-retroviral drugs, tricyclic anti-depressants, anti-tuberculosis, steroids. ACEI: angiotensin converting enzyme inhibitors (enalapril, Lisinopril, captopril), diuretics (furosemide, hydrochlorothiazide), CCB: calcium channel blockers (nifedipine, amlodipine), iron supplement (ferrous sulfate, ferrous gluconate, ferrous sucrose), Anti-DM: anti-diabetics (metformin, glibenclamide), BB: beta blockers (atenolol, metoprolol, carvedilol), statins (atorvastatin, simvastatin), phosphate binders (calcium carbonate).

The most common prescribed class of drugs among the study participants, were diuretics (70.2%), CCB (50%) and ACEI (37.1%) (Fig 1).

Among stage 4 patients, diuretics (79.7%), CCB (61.7%), iron supplements (45.1%), phosphate binders (42.9%), and BB (32.3%) were the most frequently prescribed drugs. In stage 3, most widely prescribed classes of drugs were ACEI (63.7%), diuretics (59.3%), and CCB (36.3%). Similarly, the most common drug classes in stage 2 patients were ACEI (73.7%), diuretics (57.9%), CCB (47.4%), and statins (15.8%) (Table 3).

**Table 3:** Distribution of medication used among patients with CKD at St. Paul’s Hospital Millennium Medical College, Addis Ababa, Ethiopia, June-August, 2019.

Variables					Stage of CKD				
		Stage 1		Stage 2		Stage 3		Stage 4	
		N	%	N	%	N	%	N	%
**ACEI**	YES	5	100	14	73.7	58	63.7	15	11.3
	NO	-	-	5	26.3	33	36.3	118	88.7
**Diuretics**	YES	3	60	11	57.9	54	59.3	106	79.7
	NO	2	40	8	42.1	37	40.7	27	20.3
**CCB**	YES	-	-	9	47.4	33	36.3	82	61.7
	NO	5	100	10	52.6	58	63.7	51	38.3
**Iron sup**	YES	1	20	1	5.3	18	19.8	60	45.1
	NO	4	80	18	94.7	73	80.2	73	54.9
**Epoetin**	YES	-	-	-	-	-	-	12	9.02
	NO	5	100	19	100	91	100	121	90.98
**Anti DM**	YES	1	20	2	10.5	10	10.99	8	6.01
	NO	4	80	17	89.5	81	89.01	125	93.99
**BB**	YES	-	-	2	10.5	14	15.4	43	32.3
	NO	5	100	17	89.5	77	84.6	90	67.7
**Statin**	YES	1	20	3	15.8	31	34.1	37	27.8
	NO	4	80	16	84.2	60	65.9	96	72.2
**Vit D**	YES	-	-	-	-	5	5.5	20	15
	NO	5	100	19	100	86	94.5	113	85
**Insulin**	YES	-	-	1	5.3	10	10.99	32	24.1
	NO	5	100	18	94.7	81	89.01	101	75.9
**Phosphate binder**	YES	-	-	2	10.5	12	13.2	57	42.9
	NO	5	100	17	89.5	79	86.8	76	57.1
**others**	YES	-	-	2	10.5	46	50.5	70	52.6
	NO	5	100	17	89.5	45	49.5	63	47.4

### Prevalence and types of medication related problems

**Fig 2:**
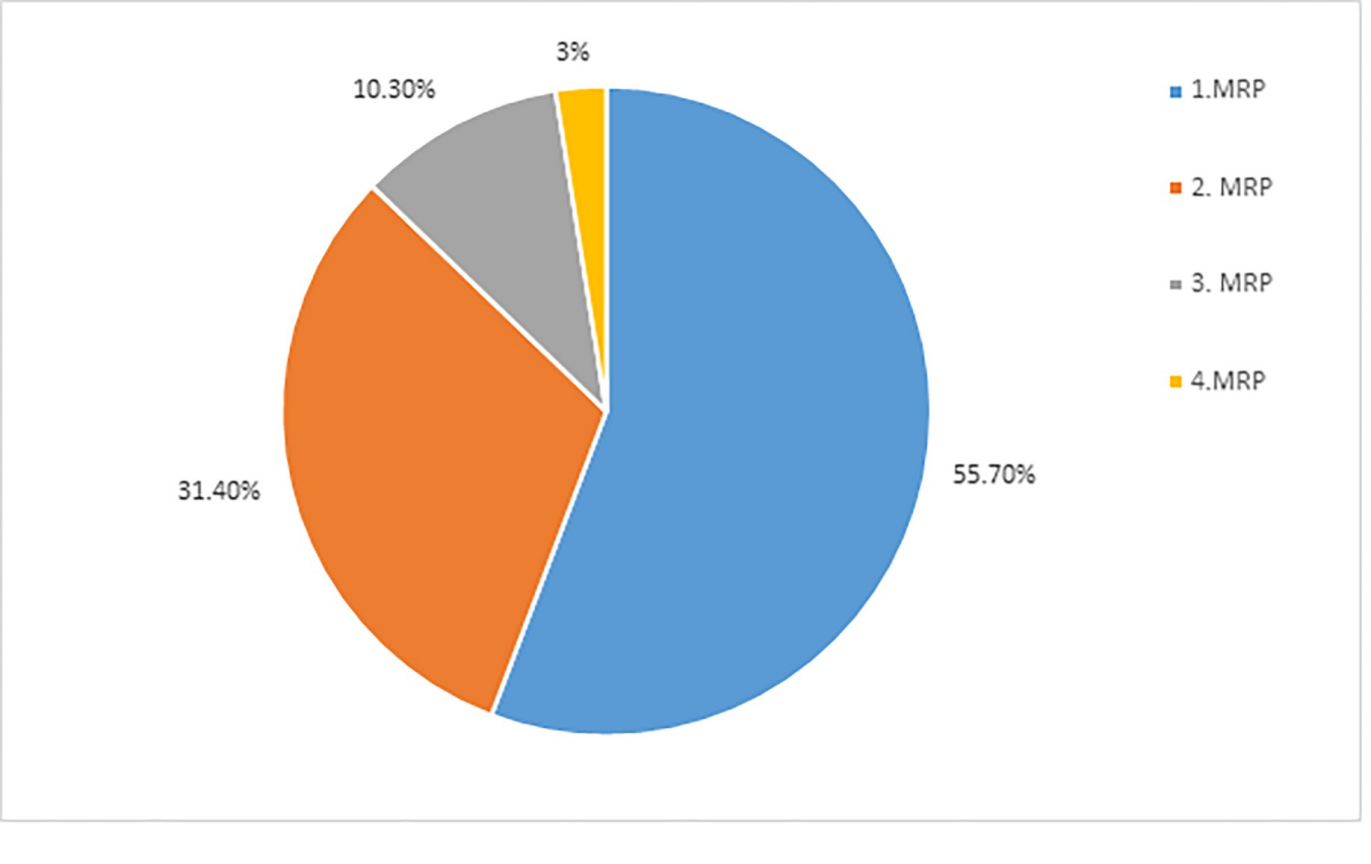
Number of medication related problems per patients among CKD patients having follow-up at renal clinic of St’s Paul’s Hospital Millennium Medical College, Addis Ababa, Ethiopia, June -August, 2019.

A total of 325 MRPs were identified from 204 (82.3%) study participants, giving 1.6 MRPs per participant. One MRP was identified in 114 (55.7%) patients and two MRPs in 64 (31.4%). The common classes of MRPs identified were; need additional drug therapy problems 114 (35.1%), non-compliance 54 (16.6%) and ineffective drug therapy 37 (11%) (Fig 2).

### Types and causes of MRP identified

The most frequently reported MRPs were the need additional drug 114 (46%), non-compliance 54(21.8%), unnecessary drug use 46(21.8%) and dose too low 46(21.8%) were the most frequently reported MRPs. The most common identified cause of need additional drug therapy were need of preventive drug therapy 80 (70.2%), and presence of untreated condition 27 (23.7%). The two most common reported causes of non-compliance were unaffordability of drugs 26(48.1%) and lack of patient understanding about drug instruction 10 (18.5%). Of 46 unnecessary drug therapy problems, 16(34.8%) were due to patients using medicine as recreation and 18 (39.1%) were due to medicines used for treating side effects of other drugs (Table 4).

**Table 4:** Types of and causes of MRP identified among patients with CKD at St. Paul’s Hospital Millennium Medical College, Addis Ababa, Ethiopia, June-August, 2019.

MRP category	Cause of MRP	Number	Percent (%)
**Unnecessary drug therapy(n = 46)**	Medicine for recreation	18	39.1
	Medicine for treating side effect	16	34.8
	Drugs without indication	8	13.4
	Duplicate therapy	4	8.5
**Need additional drug therapy (n = 114)**	Preventive drug therapy needed	80	70.1
	Untreated condition	27	23.7
	Synergistic therapy	7	6.1
**Ineffective drug therapy (n = 37)**	Not the most effective drugs	24	64.9
	Condition refractory to the drug	13	35.1
**Dosage too low (n = 46)**	Drug interaction	33	71.7
	Less frequent administration	8	17.4
	Low dose	6	13
	Short duration	1	2.2
**ADR (n = 23)**	Cause undesirable effect	5	21.7
	Unsafe drugs	7	30.4
	Drug interaction	10	43.5
	Incorrect administration	1	4.3
**Dosage too high (n = 5)**	High dose	4	80
	More frequent	1	20
**Non-compliance (n = 54)**	Drug product too expensive/ Can’t afford	26	48.1
	Direction not understood	10	18.5
	Patient prefers not to take	5	9.3
	Patient forget to take	8	14.8
	Patient can’t swallow	2	3.7
	Drug product not available	3	5.6

Among a total 51 drug-drug interactions (DDI) identified, 11 (19.7%) were major DDI, while 14 (25%) were moderate and 26 (46.5%) were minor DDI (Table 5).

**Table 5:** Frequency of major drug interaction in CKD patients having follow up at renal clinic of St. Paul’s Hospital Millennium Medical College, Addis Ababa, Ethiopia, June -August, 2019.

Drug involved in DI	Frequency	Severity	Possible effect
Aspirin + Metformin	6(10.7%)	Major	Risk of hypoglycemia
Aspirin + Furosemide	4(7.1%)	Major	Reduced effectiveness
Allopurinol + Enalapril	1(1.9%)	Major	Risk of hypersensitivity
Iron sulfate + Omeprazole	5(8.9%)	Moderate	Risk of low bioavailability
Iron gluconate + Omeprazole	2(3.6	Moderate	Risk of low bioavailability
Aspirin + Calcium carbonate	4(7.1%)	Moderate	Reduced effectiveness
Aspirin + Metoprolol	3(5.4%)	Moderate	Risk of hypotension
Iron sulfate + Calcium carbonate	16(28.6%)	Minor	Risk of low bioavailability
Calcium carbonate + iron gluconate	10(17.9%)	Minor	Risk of low bioavailability

### Predictors of medication related problems

In the multivariable regression, occupation (AOR = 5.2, 95% CI: 1.292–21.288, P = 0.020) and ACEIs use (AOR = 6.6, 95% CI: 2.202–19.634, P = 0.001) were significantly associated with the occurrence of MRP. Thus, unemployed individuals were about five times more likely to have MRP as compared with employed ones. On the other hand, patients who were not using ACEIs were seven times more likely to have MRP compared to those using ACEIs (Table 6).

**Table 6:** Predictors of MRPs among CKD patients having follow up at renal clinic of St’s Paul’s Hospital Millennium Medical College, Addis Ababa, Ethiopia, June -August, 2019.

Variables	Category	MRP		COR	AOR	P- value
		Yes	No			
Occupation	Unemployed	86(42.2%)	14(31.8%)	1.8(0.498–6.828)	5.2(1.292–21.288)	0.020*
	Employed	84(41.2%)	27(61.4%)	1	1	
Stage	1	3(60%)	2(40%)	1	1	
	2	13(68.4%)	6(31.6%)	8.2(1.224–54.919)	0.7(0.071–6.861)	0.758
	3	65(71.4%)	26(28.6%)	5.7(1.775–18.152)	0.9(0.196–4.124)	0.891
	4	123(92.5%)	10(7.5%)	4.9(2.236–10.828)	1.1(0.388–3.232)	
Co morbidity	Absent	22(66.7%)	11(33.4%)	1	1	
	Present	182(84.5%)	33(15.5%)	0.4(0.161–0.818)	0.8(0.255–2.481)	0.694
Complication	Absent	61(69.3%)	27(30.7%)	1	1	
	Present	143(89.4%)	17(10.6%)	0.3(0.137–0.528)	1.2(0.419–3.461)	0.729
ACEI use	No	147(72.1%)	9(20.5%)	1	6.6(2.202–19.634)	0.001*
	Yes	57(27.9%)	35(79.5%)	10(4.534–22.182)	1	
No of medication	≤ 3	86(74.8%)	29(25.2%)	1	1	
	≥ 4	118(88.7%)	15(11.3%)	2.7(1.341–5.249)	1.1(0.434–2.760)	0.849

## Discussion

Patients who have CKD are at high risk of MRPs due to the presence of comorbidity, complications and complexity of the drug regimen. Presence of MRPs are of a major concern in health care delivery system due to an increased burden in cost, morbidity and mortality. Identifying MRPs and factors that contributes to their coexistence is a critical step in preventing the problems and improving the health outcome.

In this study, a total of 922 medications were used, with a mean of 3.7 drugs per patient, which was comparable with other study results [20–22]. But finding of this study was different from a study conducted in Kenya, where a mean of 4.9 drugs per patient were prescribed. This discrepancy could be due to the fact that patients included the Kenyan study were only stage 3 and 4, which was most likely to have more comorbidities as well as complications and need a complex regimen.

This study showed that the most commonly prescribed class of drug was diuretics (70%). The finding was higher than a study conducted in India (8.2%) [21]. However, small number of diuretics use (11.9%) was reported in a study conducted in Nigeria [23].

About 82.3% of study participants had MRP with 1.6 MRPs per patient. This finding was in line with a study conducted in Jimma, which was 78.6% with an average of 1.9 MRP per patient [24]. The prevalence of MRPs reported in the present study was lower than a study conducted in Kenya in which 100% of the study participants had MRP [25]. The discrepancy could be due to differences in classification of MRPs, nature of study participants (only patients with CKD stage 3 and 4 were included in the Kenyan study). A higher prevalence (100%) of MRPs was also reported in studies conducted in Indonesia and India [7, 22]. The variation could be due to the nature of the study participants (admitted patients undergoing dialysis were included). Moreover, in the Indonesian study more than 80% of the study participants were in stage 5 and different MRPs classification was used which could increase the prevalence of MRPs. However, this result was higher than other studies done in Nigeria, which was 70% [26]. The differences could be attributed to difference in study design (cross-sectional vs prospective interventional), and in the stage of CKD patients (in the Nigerian study majority of study participants were at an early stage of CKD in contrast to the present study).

The commonest MRP encountered in the current study was the need additional drug therapy (35.1%). The finding was consistent with studies conducted in France (32%) (2) and Jimma (31%) [24]. However, a study conducted in Kenya reported that only 18% of indication without drugs [27]. This discrepancy might be due to fear of Physicians regarding the toxicity effect of drugs, small sample size, and differences in health care practices.

The second most frequently identified type of MRP was non-compliance (16.6%). Around half of non-compliance was due to unaffordability (48%) of the prescribed medications, followed by lack of understanding about drug instructions. These findings can be attributed to the failure of the patients to understand their disease process and the merit of compliance to medications as prescribed. A similar result was reported in a study conducted in Indonesia (19%) [7]. But a lower finding was reported from a study conducted in Nigeria (4.7%) [19]. This might be due to presence of pharmacists’ services in the setup and low pill burden.

The other MRP identified was unnecessary drug therapy (14.2%). Approximately 39% of unnecessary drug therapy was due to patients’ use of medicine for recreation. Proton pump inhibitors and analgesics were commonly prescribed without clear indication. This finding was closely related to studies conducted in France 12.8% [26], Indonesia 15% [7] and Nigeria (12.2%) [25]. However, the prevalence of unnecessary drug therapy in India was 9.5% [22]. Such variation might be due to the study design (prospective interventional versus cross-sectional) and the fact that the Indian study didn’t include social drugs as unnecessary drug therapy which in turn lower the prevalence.

In the current study, dosage too low accounted 14.2% of all MRPs which was commonly due to drug interactions. A drug commonly identified with dosage too low was iron supplement which was in line with studies conducted in France [26] and Jimma [24].

Ineffective drug therapy (11%) was among commonly identified MRP. The major causes of the problem were the selection of less effective drugs and drug interactions which was in line with a study conducted in Jimma 10% [24]. But a higher prevalence of ineffective drug therapy was reported in Iraq 27.7% [28]. This difference might be because patients with end stage renal failure on long-term hemodialysis were recruited in the Iraqi study.

In this study adverse drug reactions was reported among (7.1%) of the patients. More than 40% of them were due to drug interactions and usage of unsafe drugs. A similar finding (9%) was also reported in Nigeria [25]. Conversely, around 19% of ADR was reported in India [22]. The possible explanation could be the fact that the study in India was carried over a nine month period, thus providing sufficient time for identifying more ADRs over time and patients on dialysis who were prone to ADR were recruited in the study. In comparison, this finding was lower than the studies conducted in the USA (20.7%) [29].

A total of 51 drug-drug interactions were identified in the present study. This was lower than the findings from India 365 drug-drug interactions [30] and in Nigeria (898) [23]. This difference could be the two studies were conducted prospectively for a long time, which will increase the detection of the drug interaction. Moreover, the study participants in the previous study were elderly with a high rate of dialysis and ploy-pharmacy that might increase the risk of drug interactions.

Identification of the risk factors for MRPs is important to identify the most susceptible patients and to provide a close monitoring of drug therapy. This study found a statistical link between occupation and ACEI use and the occurrence of MRPs, which differs from most previous studies [7, 31]. Marital status, polypharmacy, number of complications, number of co morbidities and stage of CKD were the predictors of the occurrence of MRP in studies conducted in Jimma and Kenya. This discrepancy might be due to differences in health care delivery systems, socio-demographics and stage of CKD among the study participants.

### Limitation of the study

The study was a cross-sectional study that didn’t allow us to follow patient conditions prospectively. When identifying ADR, no causal relationships were established; rather, it was retrieved from medical records and patients interviews.

## Conclusion

The study revealed that MRPs experienced by the study participants was high. Need additional drug therapy was the most frequently identified type of MRP, followed by non-compliance, ineffective drug therapy and dosage too low. The majority of need additional drug therapy problems were due to absence of preventive drug therapy whereas the unaffordability of medication was the main cause for non-compliance. In the other hand selection of less effective drugs was the common cause for ineffective drug therapy problems. The most common cause of ADR and low dosage was drug interaction. There was a significant association between occupation and ACEI use with the occurrence of MRP. Hence, the provision of pharmaceutical care for early detection, prevention, and resolution of MRPs. Moreover, there should be a patient education program to improve compliance to their medication. Further studies with a follow-up of patients with intervention should be considered.

## Supporting information

S1 File Data collection tool. (DOCX)

S2 File Raw data. (SAV)

## Abbreviations

CKD: Chronic Kidney Disease
CKDUC: CKD Unknown
CRF: Chronic Renal Failure
eGFR: Estimated Glomerular Filtration Rate
ESRD: End stage renal disease
DRP: Drug Related Problem
GFR: : Glomerular Filtration Rate
MRPs: : Medication Related Problems
