# Supplementary material for: Medication related problems among ambulatory patients with chronic kidney disease at St. Paul’s Hospital Millennium Medical College, Addis Ababa, Ethiopia
Source: PLoS One. 2022 Dec 1;17(12):e0278563. doi: 10.1371/journal.pone.0278563 (PMC9714937; doi:10.1371/journal.pone.0278563)
Supplement: S1 File — (DOCX) [file pone.0278563.s001.docx]

# Data collection tool

**Section A: Patient Survey (interviewing and chart review)**

**Code of Participant**....................

1. **Socio demographic Data**
2. Age -----------------------
3. Gender : Male Female
4. Marital Status:

Single separated married divorced widowed

1. **Family History**
2. What is the patient’s relevant family history? State briefly below

................................................................................................................................................................................................................................................................................................

1. **Social History**
2. What is the patient’s social history? Please fill in the space below

| Occupation | Unemployed employed retire |
| --- | --- |
| Income/month | <500 500-1000 1000-3000 3000-6000 >6000 |
| Education | Illiterate primary secondary college and more |
| Religion | Orthodox Muslim catholic protestant |
| Alcohol intake | Yes No |
| Smoking | Yes No |

1. **MRP reported by the patients**
2. Does the patient have trouble using his/her medicine? Yes No

If yes, briefly state their concern below

................................................................................................................................................................................................................................................................................................................................................................................................................................................

1. Does the patient have trouble in understanding or remembering how to take his/her medicine? Yes No

If yes, please list the problems they encounter in the space below

................................................................................................................................................................................................................................................................................................

1. Do any patient’s medication make him/her feel unwell? Yes No

If yes, please indicate how they feel when they take medicine below

................................................................................................................................................................................................................................................................................................

1. Does he/she sometimes stop taking his/her medicine? Yes No

If yes, please indicate when they cease, lead them to stop taking medicine

................................................................................................................................................................................................................................................................................................................................................................................................................................................

1. Does the patient feel like he/she is taking too many drugs? Yes No

If yes, please indicate their major concern in the space below

................................................................................................................................................................................................................................................................................................

1. Does the patient feel like the medicine he/she is taking is making him/her feel better? Yes No

If No, please indicate what they feel and which medicine make them feel that way in the space below

................................................................................................................................................................................................................................................................................................

1. Does the cost of patient’s medicine make it hard for his/her to take it as prescribed?

Yes No

If yes, please state which medicine they have missed because they find them expensive below

...............................................................................................................................................................................................................................................................................................

1. **Review of Systems**
2. **Chief Complaint**
3. What is the patient’s chief complaints? State briefly below

................................................................................................................................................................................................................................................................................................................................................................................................................................................

1. **History of Present Illness**
2. What is the patient’s history of present illness? State briefly below

................................................................................................................................................................................................................................................................................................................................................................................................................................................

1. **Past Medical History**
2. What is the patients past medical history? State briefly below

................................................................................................................................................................................................................................................................................................

1. **Medication History**
2. What is the patients past medical history? Please conduct comprehensive past medication history and fill in the table below

| Medicines /dose | Indication | Duration (date) | | Comment (outcmes,allergy.ADR) |
| --- | --- | --- | --- | --- |
|  | | Start | Stop |  |
| **Allergy:** | | | | |
| **Current medication history**: prescription and non-prescription | | | | |
|  |  |  |  |  |
|  |  |  |  |  |
|  |  |  |  |  |
|  |  |  |  |  |
|  |  |  |  |  |
|  |  |  |  |  |
|  |  |  |  |  |
| **Past medication history:** prescription and non-prescription | | | | |
|  |  |  |  |  |
|  |  |  |  |  |
|  |  |  |  |  |
|  |  |  |  |  |
|  |  |  |  |  |
|  |  |  |  |  |
|  |  |  |  |  |
| **Home remedies/herbal preparation/dietary supplements** | | | | |
|  |  |  | |  |
|  |  |  | |  |
|  |  |  | |  |
|  |  |  | |  |

1. Please conduct a comprehensive review of systems. Are there any significant findings on the review of systems in relation to pharmacotherapy?

Yes No

If yes, briefly clarify it below

................................................................................................................................................................................................................................................................................................................................................................................................................................................................................................................................................................................................

1. **Investigation**
2. Have any investigation been carried out? Please fill the result below

| Test/investigation | Date carried out | Value | Comment (normal vs abnormal) |
| --- | --- | --- | --- |
| **Vitals** | | | |
| HR |  |  |  |
| BP |  |  |  |
| RR |  |  |  |
| Body T^o^ |  |  |  |
| **Blood analysis** | | | |
| RBC |  |  |  |
| Hb |  |  |  |
| MCV |  |  |  |
| WBC |  |  |  |
| Neutrophils |  |  |  |
| Lymphocytes |  |  |  |
| Monocytes |  |  |  |
| Eosinophils |  |  |  |
| **Coagulation** | | | |
| Prothrombin time |  |  |  |
| APTT |  |  |  |
| INR |  |  |  |
| **Electrolyte** | | | |
| Na^+^ |  |  |  |
| K^+^ |  |  |  |
| Mg^2+^ |  |  |  |
| Cl^-^ |  |  |  |
| Urea |  |  |  |
| Cr |  |  |  |
| Clcr |  |  |  |
| Ca^2+^ |  |  |  |
| PO^-^_4_ |  |  |  |
| **LFTs** | | | |
| AST |  |  |  |
| ALT |  |  |  |
| GGT |  |  |  |
| ALP |  |  |  |
| Alb |  |  |  |
| Total bilirubin |  |  |  |
| **BGA** | | | |
| PH |  |  |  |
| PO2 |  |  |  |
| PCO2 |  |  |  |
| HCO-3 |  |  |  |
| **Blood glucose** | | | |
| RBS |  |  |  |
| FBS |  |  |  |
| HbA1c |  |  |  |
| **Others** | | | |
| CxR |  |  |  |
| ECG |  |  |  |
| Urinalysis |  |  |  |
| HIV |  |  |  |
| HBV |  |  |  |
| HCV |  |  |  |

1. **Diagnosis**
2. What is the current working diagnosis or confirmed diagnosis? Briefly state below

| **Diagnosis** | **Comment** |
| --- | --- |
| ......................................................................  ......................................................................  ......................................................................  ...................................................................... | .............................................................  .............................................................  .............................................................  ............................................................. |

**Section B: Evaluation of MRP**

1. **Prevalence of MRP**
2. Did the patient have any MRPs? Yes No

If yes, please elaborate by filling the section below

1. **Classification**
2. Please classify the MRP(s) in the categories provided below. Please justify by providing comment. Also state the medicine involved.

| Classification of MRP | Comment | Medicine involved | Therapeutic category |
| --- | --- | --- | --- |
| Unnecessary Drug Therapy |  |  |  |
| Need Additional Drug Therapy |  |  |  |
| Ineffective Drug Therapy |  |  |  |
| Dose Too Low |  |  |  |
| Adverse Drug Reaction |  |  |  |
| Dose Too Low |  |  |  |
| Non-compliance |  |  |  |

**Have you ever missed your medication used for your renal and related conditions?**

If you have any problems that challenges your medication adherence please the select your reason

- Directions not understood
- Patient prefers not to take
- Patient forgets to take
- Drug product too expensive
- Patient cannot swallow/ administer
- Drug product not available
- Patient felt better worse
- Regimen complexity
- Fear of adverse events
- Patient felt better
- Disbelief in drug effectiveness
- Others, specif-----------------------------------------------------------

Drug- Drug interaction

| Interaction | Severity | |  | | Possible effect | Remark |
| --- | --- | --- | --- | --- | --- | --- |
|  | Severe | Moderate | | Mild |  |  |
|  |  |  | |  |  |  |
|  |  |  | |  |  |  |
|  |  |  | |  |  |  |
|  |  |  | |  |  |  |
|  |  |  | |  |  |  |
|  |  |  | |  |  |  |
|  |  |  | |  |  |  |
|  |  |  | |  |  |  |
|  |  |  | |  |  |  |
|  |  |  | |  |  |  |
|  |  |  | |  |  |  |
|  |  |  | |  |  |  |
|  |  |  | |  |  |  |
|  |  |  | |  |  |  |
|  |  |  | |  |  |  |
|  |  |  | |  |  |  |
